# Supplementary material for: Compliance with 24 h Movement Behavior Guidelines for Pregnant Women in Saudi Arabia: The Role of Trimester and Maternal Characteristics
Source: Healthcare (Basel). 2024 Oct 15;12(20):2042. doi: 10.3390/healthcare12202042 (PMC11506994; doi:10.3390/healthcare12202042)
Supplement: Supplementary file 1 [file healthcare-12-02042-s001.zip › healthcare-3229558-supplementary.pdf]

Table S1. Compliance with the Individual 24-Hour Movement Behaviors Guidelines by Trimester without adjustment.

**Trimesters**	**Adhered to MPA Guideline (150 min/week)**			**Adhered to Sleep Duration Guideline (7 – 9 h/day)**			**Adhered to Total SB Guideline (< 8 h/day)**		
	**n (%)**		**PR (95% CI)**	**n (%)**		**PR 95%(CI)**	**n (%)**		**PR** 95%(CI)
	**Yes**	**No**		**Yes**	**No**		**Yes**	**No**	
**First (n=225)**	83 (36.9%)	142 (63.1)	Reference	107 (47.6%)	118 (52.4%)	Reference	154 (68.4%)	71 (31.6%)	Reference
**Second (n=317)**	165 (52.0%)	152 (48.0%)	**1.41 (1.15 – 1.73)**	176 (55.5%)	141 (44.5%)	1.17 (.99 – 1.38)	222 (70.0%)	95 (30.0%)	1.01 (.91 – 1.13)
**Third (n=393)**	163 (44.0%)	524 (56.0%)	1.12 (.91 – 1.38)	227 (57.8%)	166 (42.2%)	**1.21 (1.03 – 1.43)**	272 (69.2%)	121 (30.8%)	1.01 (.91 – 1.13)

CI; confident interval, MPA; moderate physical activity, n; number, *p*; p-value, SB; PR; prevalence ratio, sedentary behavior.

Table S2. Compliance with the Combined 24-hour Movement Behaviors Guidelines by Trimester without adjustment.

Trimesters	Met MPA + Sleep Duration Guidelines			Met MPA + Total SB Guidelines			Met Sleep Duration + Total SB Guidelines			Met MPA + Sleep Duration + Total SB Guidelines		
	n (%)		PR 95%(CI)	n (%)		PR 95%(CI)	n (%)		PR 95%(CI)	n (%)		PR 95%(CI)
	Yes	No		Yes	No		Yes	No		Yes	No	
**First (n=225)**	42 (18.7%)	183 (81.3%)	Reference	56 (24.9%)	169 (75.1%)	Reference	70 (31.1%)	155 (68.9%)	Reference	29 (12.9%)	196 (87.1%)	Reference
**Second (n=317)**	92 (29.0%)	225 (71.0%)	**1.48 (1.13 – 1.94)**	117 (36.9%)	200 (63.1%)	**1.55 (1.13 – 2.15)**	127 (40.1%)	190 (59.9%)	**1.29 (1.02 – 1.63)**	68 (21.5%)	249 (78.5%)	**1.66 (1.12 – 2.48)**
**Third (n=393)**	86 (21.9%)	307 (78.1%)	1.17 (.84 – 1.63)	108 (27.5%)	285 (72.5%)	1.10 (.84 – 1.46)	158 (40.2%)	235 (59.8%)	**1.29 (1.03 – 1.62)**	57 (14.5%)	336 (85.5%)	1.13 (.74 – 1.71)

CI; confident interval, MPA; moderate physical activity, n; number, *p*; p-value, PR; prevalence ration, SB; sedentary behavior.
